# Alpha-1 antitrypsin (AAT) augmentation therapy in individuals with the PI*MZ genotype: a pro/con debate on a working hypothesis

**DOI:** 10.1186/s12890-021-01466-x

**Published:** 2021-03-23

**Authors:** Igor Barjaktarevic, Marc Miravitlles

**Affiliations:** 1 Division of Pulmonary and Critical Care Medicine, David Geffen School of Medicine at University of California Los Angeles, Los Angeles, CA USA; 2 Pneumology Department, Hospital Universitari Vall D’Hebron, Vall D’Hebron Institut de Recerca (VHIR), Vall d’Hebron Hospital Campus, CIBER de Enfermedades Respiratorias (CIBERES), Barcelona, Spain

**Keywords:** Alpha-1 antitrypsin deficiency, Genotype, PI*MZ, Pulmonary disease, Chronic obstructive pulmonary disease

## Abstract

Alpha-1 antitrypsin deficiency (AATD) is a significantly under-diagnosed genetic condition caused by reduced levels and/or functionality of alpha-1 antitrypsin (AAT), predisposing individuals to lung, liver or other systemic diseases. The management of individuals with the PI*MZ genotype, characterized by mild or moderate AAT deficiency, is less clear than of those with the most common severe deficiency genotype (PI*ZZ). Recent genetic data suggest that the PI*MZ genotype may be significantly more prevalent than currently thought. The only specific treatment for lung disease associated with severe AATD is the intravenous infusion of AAT augmentation therapy, which has been shown to slow disease progression in PI*ZZ individuals. There is no specific evidence for the clinical benefit of AAT therapy in PI*MZ individuals, and the risk of emphysema development in this group remains controversial. As such, current guidelines do not support the use of AAT augmentation in PI*MZ individuals. Here, we discuss the limited data on the PI*MZ genotype and offer *pro* and *con* perspectives on pursuing an AAT-specific therapeutic strategy in PI*MZ individuals with lung disease. Ultimately, further research to demonstrate the safety, risk/benefit balance and efficacy of AAT therapy in PI*MZ individuals is needed.

## Background

Most of the published evidence relating to the management of individuals with alpha-1 antitrypsin deficiency (AATD) is based on patients with the PI*ZZ or PI*Znull genotypes, who have a severe deficiency in alpha-1 antitrypsin (AAT), with plasma levels < 11 µM (< 52 mg/dL) compared with the normal range of 19–47 µM (102–254 mg/dL) [1]. Nevertheless, there are an estimated 6–7 million people with AATD in the United States (US) alone, including those with mild or moderate deficiency genotypes [2]. Individuals who are heterozygous for the Z allele, such as those with the PI*MZ genotype, who have AAT serum levels of 11–28 µM (62–151 mg/dL), or approximately 60% of the normal range [1], may be at risk of developing lung and/or liver disease if they have other predisposing risk factors. Previous studies have found that among patients diagnosed with chronic obstructive pulmonary disease (COPD), the prevalence of the PI*MZ genotype ranges from 1 to 22% [3, 4].

Despite the understanding of the mechanisms responsible for pathologic changes in AAT-deficient individuals [5], and the fact that AAT augmentation therapy is the only disease-modifying therapeutic approach for patients with AATD-associated lung disease [6], current guidelines do not recommend the use of augmentation therapy in individuals with the PI*MZ genotype. However, there is a lack of consensus among treating physicians on how PI*MZ individuals should be monitored and treated, and whether AAT augmentation therapy could be a treatment strategy in some PI*MZ patients. Here, we summarize the evidence supporting a potential AAT-specific therapeutic approach in individuals with the PI*MZ genotype, and also discuss reasons why focus on this approach may not be warranted.

## Main text

### Pro: AAT treatment of PI*MZ patients could be an option

#### Focus on AAT serum concentrations in PI*MZ can be misleading

1) The relevance of the *‘protective threshold’*

Systemic levels of AAT in PI*MZ individuals do not generally fall below 11 µM, a value historically used as a theoretical ‘protective threshold’ for AAT therapy provision; levels below this threshold are thought to be associated with a higher risk of developing emphysema [7]. There are, however, several issues with this theoretical threshold as a reason to treat or withhold AAT augmentation therapy. The *‘*protective threshold’ of 11 µM was chosen based on historical data drawn from standards that lacked adequate accuracy and clinical validation [7, 8]. The threshold represents a systemic concentration of AAT in serum rather than its level in pulmonary epithelial lining fluid, and thus, does not necessarily accurately reflect AAT functional activity in the lung microenvironment. Furthermore, this threshold concentration is based on nephelometric measurement of antigenic, rather than functional, activity, which may be lower due to misfolded, dysfunctional proteins in some individuals [9, 10]. Normalizing AAT serum levels in patients with severe AATD with doubling the standard dose of augmentation therapy resulted in a significant reduction in inflammatory markers compared with traditional dosing [11], further questioning the approach to using a threshold value to dichotomize the problem and determine the management. At the same time, the PI*MZ phenotype is associated with a wide range of abnormal AAT levels. While the 2003 European Respiratory Society/American Thoracic Society statement indicates that AAT levels are only mildly reduced in those with the PI*MZ phenotype (17–33 µM, or 90–210 mg/dL) [3], two screening studies reported much lower levels, with similar ranges of ~ 11.9 to 29.0 µM (62–151 mg/dL) [12] and ~ 12.7 to 19.2 µM (66–100 mg/dL) [13]. In addition, it is known that heterogeneity of disease exists independent of AAT serum levels. For example, PI*ZZ individuals who have reduced serum levels may be asymptomatic or have mild symptoms of lung disease, while others may have severe lung disease [14], suggesting that the relationship between AAT serum levels and presentation of disease may not always be predictable.

Although there are substantial limitations to the ‘protective threshold’, it should be noted that a solid alternative to this does not exist today. A shift toward a model of AAT deficiency based on functionality—rather than quantity—is warranted; however, quantitative AAT serum assays are widely used and inexpensive, while assays assessing AAT functional activity [15] are utilized in only a handful of specialized laboratories globally. Therefore, progress towards an inexpensive, reproducible, and widely available AAT activity assay is required.

2) Steady-state versus pro-inflammatory acute settings

The traditional evaluation of steady-state serum levels may lead to misinterpretation of its findings in two different ways. Inducible plasma levels of AAT increase in comparison to steady-state levels [16–18], and as an acute phase reactant, AAT contributes in limiting local and systemic inflammation [19]. The anti-inflammatory and cytoprotective effects of AAT are of high importance in acute inflammatory conditions, where increased levels of neutrophil elastase (NE) lead to compromised lung permeability and induce the release of pro-inflammatory cytokines [19, 20]. Thus, adequate immune response in acute inflammation may be, to a certain extent, dependent on appropriate AAT increase, which is compromised in patients with AATD, including heterozygous PI*MZ individuals. On the other hand, unrecognized inflammation at the time of presumed “steady-state” measurement of AAT levels can mask the actual level of AAT deficiency and lead to overestimation of baseline AAT levels, compromising the ability to identify PI*MZ individuals at risk of progressive lung function deterioration [21]. Data from a national AAT deficiency-targeted screening cohort showed that approximately a quarter of PI*MZ samples showed signs of inflammation, as evidenced by increased levels of C-reactive protein (CRP) ≥ 5 mg/L [21]. This indicates that the actual ‘steady-state’ levels of AAT in these individuals when inflammation is not present may be lower than measured. In addition, AAT and CRP serum levels are elevated in non-deficient COPD subjects, suggesting an increased level of systemic inflammation in COPD and that increased levels of AAT may be a physiologic response to compensate for this increased inflammation [22].

3) AAT levels versus protease-antiprotease balance

**Fig. 1:**
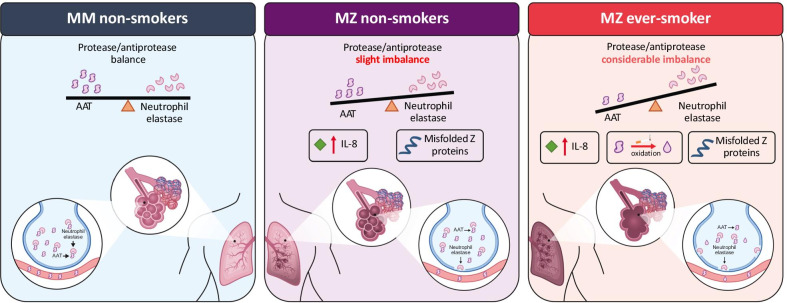
Potential mechanism for increased disease risk in individuals with the PI*MZ genotype. Left panel: In MM non-smokers, a normal protease/antiprotease balance exists with normal alveoli. Central panel: MZ non-smokers have a slight imbalance of AAT and neutrophil elastase, IL-8 levels may increase in the lungs causing neutrophil activation and inflammation and progressive damage in the lungs in some patients. Right panel: MZ smokers have a greater imbalance between AAT and neutrophil elastase as a result of reactive oxygen species in cigarette smoke inactivating AAT. Additional factors such as Z polymers and IL-8 inflammatory markers cause greater production of neutrophil elastase in the lung, causing irreversible damage. Figure adapted from Carroll et al. 2014; https://doi.org/10.5772/58602 [23] under the Creative Commons Attribution 3.0 License. *AAT* alpha-1 antitrypsin, *IL* interleukin

While we acknowledge the relevance of measuring and interpreting serum AAT levels, the ultimate goal of AAT augmentation therapy is to restore the balance of proteases and antiproteases in these patients in the long term. PI*MZ individuals generally have a more favorable protease-antiprotease balance than PI*ZZ individuals due to higher levels of AAT. Nevertheless, the deficiency of functional AAT in PI*MZ individuals, and the accumulation of misfolded/non-functional AAT, may increase inflammation in the lungs due to the reduced inhibition of NE and increased chemoattractant production, that could facilitate neutrophil activation and increase enzyme activity, exacerbating lung disease in some individuals (Fig. 1) [23]. It is also under-appreciated that AAT inhibits proteases other than NE, such as proteinase-3, and has important immunomodulatory functions [11, 24, 25], interruption of which may also be disease-causing. Infusions of AAT in severely deficient individuals reduces leukotriene B4 (LTB4) and NE activity in the lung, as well as reducing a range of pro-inflammatory cytokines [11, 26]. Sputum analysis of PI*MZ subjects without airflow obstruction identified inteleukin-8 (IL-8)-related neutrophilic inflammation in the airways, similar to stable COPD patients, suggesting an increased risk of progressive pulmonary changes related to the pro-inflammatory consequences of raised neutrophil levels in individuals with reduced functional AAT [27]. Furthermore, the formation of Z polymers, particularly at the sites of inflammation, could amplify the immune response and may cause further damage leading to emphysema [28].

The imbalance between AAT and proteases may not only be related to AAT levels; another factor impacting disease risk in PI*MZ individuals is the genetics underpinning the expression of NE. A recent study in AATD and control subjects explored the expression of the neutrophilic elastase-encoding gene (Elastase, Neutrophil Expressed [ELANE]), which provides another indication of the degree of proteinase and antiprotease balance [29]. Expression of ELANE was found to be greatly variable, with the highest levels shown in PI*MM controls. In subjects with the PI*ZZ genotype, ELANE expression was shown to correlate with lung function, suggesting that in individuals with low AAT levels, ELANE expression is an additional modifier of protease/antiprotease imbalance and disease risk [29]. Therefore, theoretically, PI*MZ individuals with high levels of ELANE expression and reduced AAT levels may have significant protease/antiprotease imbalance and increased risk for developing COPD in comparison to PI*MZ individuals with normal ELANE or even PI*ZZ individuals with lower levels of ELANE. Focus on correcting the balance between proteases and antiproteases rather than the focus on actual levels of AAT may explain possible beneficial effects of AAT augmentation therapy in non-deficient individuals with cystic fibrosis and pneumonia, where AAT inhalation led to elastase inhibition and a reduction in the downstream effects of inflammation [25]. Following a similar concept, ongoing studies are evaluating the benefits of the inhibition of NE activity in AAT-deficient patients who are not treated with augmentation therapy [24, 25, 30].

#### PI*MZ individuals develop disease as a consequence of AATD

Several case–control studies indicated a higher prevalence of the PI*MZ genotype among patients with COPD compared with controls without COPD [31–33]. A recently published analysis of a large cohort of individuals with COPD showed that PI*Z heterozygotes with a significant cigarette smoking history are at an increased risk of COPD compared with ever-smoker PI*MM individuals, have lower lung function, greater airflow obstruction and greater computed tomography (CT)-based quantitative measures of emphysema [34, 35]. These reports suggest that PI*MZ patients have a higher susceptibility to smoking-related lung disease. Nevertheless, similar to the fact that not all PI*ZZ individuals suffer from clinically significant lung or liver disease during their lifetime, it is evident that many PI*MZ individuals do not suffer from extensive lung or liver disease.

#### Rationale for augmentation therapy in non-severely AAT-deficient individuals

Preventing lung function decline is one of the key goals in the management of COPD, and data show that forced expiratory volume in 1 s (FEV_1_) decline and exacerbation rates are significantly associated with outcomes in this disease [36]. Current guidelines recommend AAT augmentation therapy in patients with severe AATD (ZZ or Null genotypes) with airflow obstruction [37–39], with additional criteria to select the treatment candidates, including smoking cessation, FEV_1_ ≤ 65% and AAT serum levels ≤ 11 µM [38, 40]. In common practice, FEV_1_ decline [38] or exacerbation frequency [41] may influence the decision to consider initiation of the augmentation therapy in patients with severe AAT deficiency, even before all other criteria for the initiation of AAT are fulfilled [42].

In patients with severe AATD, “rapid decliners” can have an FEV_1_ loss of over 200 mL/year [43, 44]. Analysis of two independent studies found a 3.9% lower FEV_1_/forced vital capacity (FVC) ratio in PI*MZ compared with PI*MM individuals, after adjusting for pack-years, age, sex, and height, suggesting that some patients with the PI*MZ genotype may have a slight increased risk of developing AATD-related COPD compared with PI*MM individuals [45]. Understanding tremendous heterogeneity in the rate of FEV_1_ decline and survival, despite administration of recommended management approaches in COPD [36], it is clear that a prevalent population of PI*MZ individuals with COPD [3, 4, 35] includes individuals with progressive disease despite other management strategies being adequately applied. Observational evidence suggests that AAT augmentation therapy may be particularly effective in patients with severe AATD [44], and this may provide a basis to consider treating patients with heterozygous genotypes (such as PI*MZ patients) and rapid lung function decline with AAT. When investigating heterogeneity in lung function decline in PI*MZ individuals, it is important to acknowledge lack of sensitivity of routine spirometry to capture emphysema progression in patients with AATD [7], and additional tests such as diffusing capacity and quantitative imaging may be required in future studies.

From a practical standpoint, recent data suggest that a significant number of individuals in the US diagnosed with the PI*MZ genotype are prescribed AAT therapy, contrary to current indications. In an analysis of the AlphaNet disease management and prevention program (ADMAPP), out of a total of 3506 individuals, the majority of whom were receiving AAT therapy (actual numbers not specified), ~ 13% of patients were reported to have the PI*MZ genotype [46]. While we may speculate that a significant number of these individuals may have been started on this therapy simply due a lack of adherence to the guidelines, and recognize that treatment is against recommendations, the number of PI*MZ patients receiving AAT therapy in the US may provide a platform for retrospective studies on the clinical efficacy of augmentation therapy in this patient population.

#### Ethical considerations

There is an overall consensus that current recommendations to only treat severe AATD are, to certain extent, based on the lack of evidence for the clinical benefit of augmentation therapy in a larger population of AATD patients beyond the PI*ZZ genotype. However, given that there is good evidence for efficacy in an adjacent group, ethical questions arise in terms of not exploring treatment of PI*MZ patients who have a clinical state that mirrors that of PI*ZZ individuals. Furthermore, it is known that not all PI*ZZ individuals receiving AAT augmentation benefit from therapy, and in the same way, although not all PI*MZ may benefit from augmentation therapy, a proportion of patients may have improved clinical outcomes.

In addition, the consensus to only treat PI*ZZ individuals has resulted in phenotypes other than PI*ZZ or PI*Null being less adequately screened for, and may have contributed to a situation where specific interventions for less severe forms of AATD have not been well explored. This may have been exacerbated by not investigating ‘proof-of-concept’ for efficacy of AAT therapy in PI*MZ individuals, possibly slowing progression towards a more cost-effective treatment with a similar mechanism of action.

#### Conclusions

AAT is a powerful protein with multiple immunomodulatory functions. Its deficiency—severe, as seen in PI*ZZ or PI*Null individuals, or moderate, as most often seen in PI*MZ individuals—represents an abnormal state that leads to a compromised response to inflammation and predisposition to a disease state. A portion of PI*MZ individuals who, despite lifestyle modifications and elimination of risk factors with adequate non-specific treatment regimens, continue to have significant lung function deterioration, could benefit from the treatment of this genetic disorder, and AAT augmentation therapy might be an option in their management. Further research on this topic is warranted.

### Con: PI*MZ individuals should not be treated with AAT augmentation therapy

#### Disease risk and severity in patients with the PI*MZ genotype

**Fig. 2:**
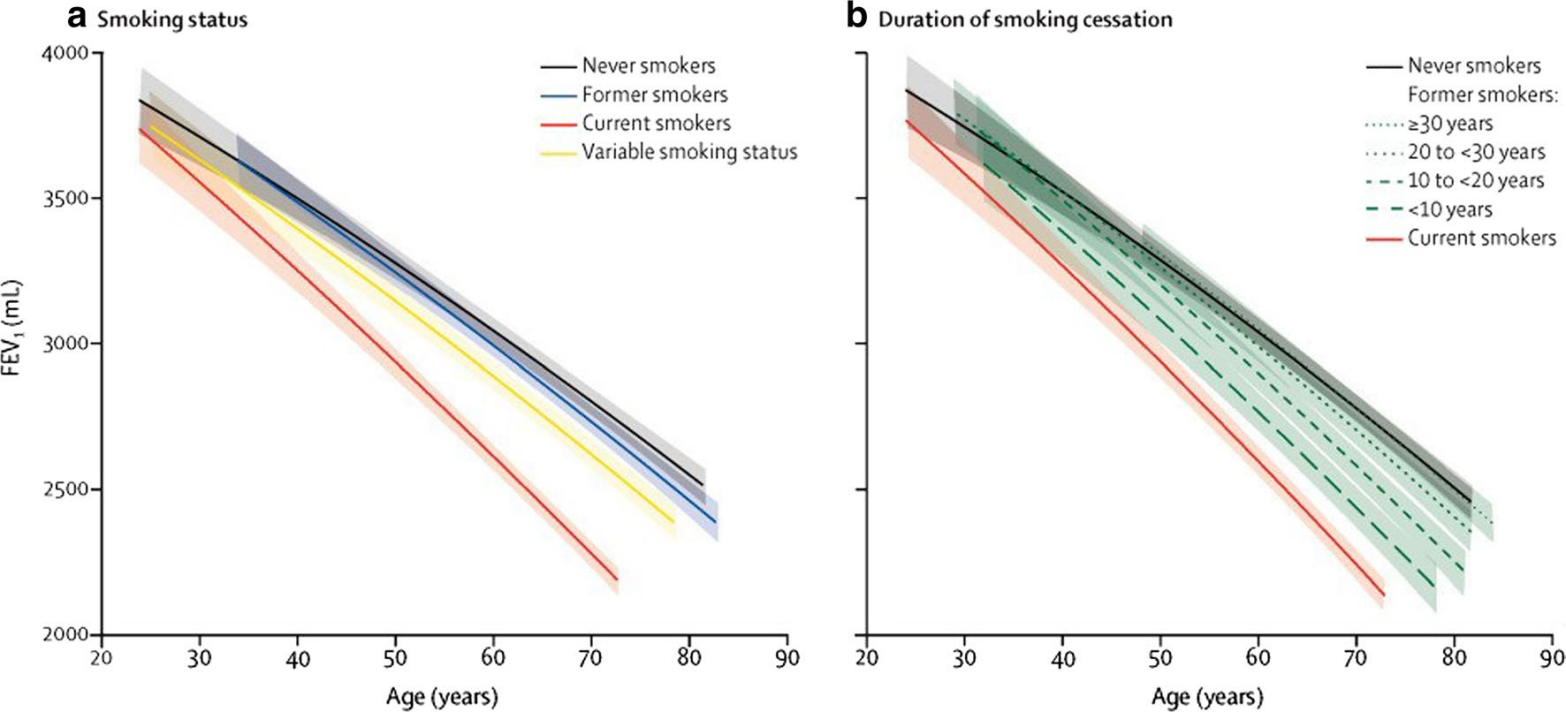
Predicted FEV_1_ curves according to smoking status (**a**) and duration of smoking cessation (**b**) [48]. Figure reproduced with permission from Oelsner et al. *Lancet Respir Med* 2020;8:34–44, Copyright Elsevier 2020. *FEV*_1_ forced expiratory volume in 1 s

It is well known that smoking is a key risk factor for the development of lung disease in patients with AATD, and disease progression and survival are both significantly worse in smokers than never-smokers [3]. This also applies to those with the PI*MZ genotype, with a family-based study showing that cigarette smoke exposure influenced the risk for impaired lung function and COPD, while PI*MZ individuals who had never smoked did not develop lung disease [35]. Furthermore, this study found that PI*MZ smokers have a higher risk of COPD in comparison to PI*MM smokers [35]. Interestingly, this difference may be limited to those with a low smoking history. Another study found that PI*MZ individuals with a smoking history of < 20 pack years had more severe emphysema on CT scan than equivalent PI*MM individuals, but this difference was not apparent between PI*MZ and PI*MM individuals with higher levels of smoking (> 20 pack years) [45]. It is therefore thought that PI*MZ individuals have a small increased risk for COPD compared with PI*MM individuals, with a small proportion of individuals having a greater risk of developing COPD, likely as a result of additional environmental or genetic risk factors [47]. Recent data from patients with non-AATD COPD showed that all levels of smoking exposure are associated with lasting and progressive lung damage, with the decline in lung function only normalizing 20 years after smoking cessation in some patients (Fig. 2) [48]. Given the increased impact of smoking in patients with AATD, these data emphasize the importance of encouraging smoking cessation to prevent deterioration in lung function.

While the clinical course of disease for PI*MZ heterozygotes is less clear than the PI*ZZ genotype, the majority of individuals with the PI*MZ genotype, especially in the absence of additional risk factors, will not develop significant lung disease that often characterizes PI*ZZ or PI*Null genotypes. A meta-analysis, which included seven cross-sectional studies reporting lung function as a continuous outcome, found no difference in mean FEV_1_% predicted between PI*MZ and PI*MM individuals (*p* = 0.62) [47]. Furthermore, data from a longitudinal community study suggested that having the PI*MZ genotype was not a significant risk factor for an accelerated decline in FEV_1_ [49]. It has also been reported that patients with COPD and the PI*MZ genotype do not have an increased risk of COPD hospitalization, unless the patient had a first-degree relative with emphysema and the PI*ZZ genotype [50]. This study suggests that, in addition to the Z allele, other genetic or environmental factors that contribute to lung disease development remain unknown.

Exposure to occupational and environmental pollutants that cause respiratory irritation (e.g., gases/fumes used in the agricultural industry) are known to be independent risk factors for lung function impairment in PI*MZ individuals, and should be avoided where possible to help maintain good lung health [51, 52]. There is, however, accumulating evidence for an increased susceptibility to occupational exposure-related lung function decline in PI*MZ individuals who are current or ex-smokers [37, 51]. In summary, individuals with the PI*MZ genotype who are non-smokers do not have an increased risk for COPD, and only a subset of individuals may be more susceptible due to other genetic or environmental factors [53]. Avoiding risk factors such as smoking is crucial in these individuals; however, the full impact of other genetic or environmental factors needs further clarification in larger studies.

While the currently available data on disease risk in PI*MZ individuals are sourced from well-designed studies and are mostly consistent, it should be noted that a limitation is that they are largely based on FEV_1_. Although FEV_1_ is a reproducible and cost-effective endpoint, it lacks sensitivity to quantify emphysema progression. Further data, based on diffusing capacity, CT or other clinical outcomes, such as exacerbations, would help clarify disease risk in PI*MZ individuals.

#### AAT therapy: current state of the evidence

It has previously been noted that the biological rationale for the provision of AAT therapy in PI*MZ individuals is unclear; with plasma AAT levels around 60% of normal, individuals have much higher AAT levels than patients with severe disease (approximately 20% of normal) [3, 5]. Patients are therefore considered to have plasma levels considerably higher than what is currently deemed the ‘protective threshold’ (11 µM), and provision of AAT augmentation therapy for these patients would therefore appear to be unnecessary based on levels of AAT alone. A longitudinal clinical trial investigating the efficacy of AAT therapy in patients with PI*MZ AATD and significant respiratory dysfunction would be required to determine efficacy in this population. However, it is debatable whether this is warranted based on what is known regarding the disease risk in this population and the strong influence of smoking on disease progression. At this time, there are no data supporting the use of AAT therapy in patients with the PI*MZ genotype (which is reflected in current guidelines/position statements [37, 38, 40]) and limited rationale to pursue this as a therapeutic approach in the future. Once we have a wider agreement of the specific phenotypic characteristics related to non-severe PI*MZ, and whether AAT deficiency is a cause or contributing factor to development of chronic lung disease, the appropriateness of the use of available specific therapies can be evaluated.

#### Ethical considerations

It is important to consider that the PI*MZ genotype is much more common than the PI*ZZ genotype, with a worldwide prevalence of 6.2% versus 0.02%, respectively [54]; therefore, it could be argued that the high prevalence of the PI*MZ genotype could potentially create shortages in the provision of AAT therapy in these patients. It is important to note that AAT is a plasma-derived protein, is in limited supply, and is costly [55]. Therefore, providing unnecessary treatment to large numbers of PI*MZ individuals poses the risk that treatment becomes less accessible for patients with more severe deficiency, who have shown a clear benefit from treatment. Although it could be argued that supply of AAT should be directed towards patients who show a response to treatment rather than based on genotype, assessing response to treatment should be ideally assessed over several years, requiring comprehensive, well-structured prospective studies. Furthermore, arguments for investigating treatment in PI*MZ individuals should be viewed in the current context, where a significant number of patients with a severe genotype eligible for treatment likely remain unidentified. Efforts should be focused on identifying and treating individuals who are most likely to benefit from augmentation therapy with the potential to change the course of disease, rather than heterozygous individuals in whom the clinical outcomes of AAT therapy are not yet clear. Moreover, focusing on the limitations of current measures, such as the precise relevance of the ‘protective threshold’, could result in a paradigm shift in terms of acceptance that current understanding of AATD is wrong, which could also cast doubt on the decision to treat homozygous individuals. Many countries/healthcare systems do not deem AAT therapy to be cost-effective and do not reimburse treatment of patients with severe deficiency, despite current recommendations [37, 42]. Furthermore, in patients with genotypes for severe disease, there are data on which to base cost-effectiveness predictions; however, there are no such data available for patients with the PI*MZ genotype. Therefore, pursuing an AAT-specific treatment strategy in patients who may not need the treatment may put an undue financial burden on healthcare systems and individuals. It is also important to consider that treatment with AAT therapy is usually lifelong. This may suggest that some patients with the PI*MZ genotype could receive unnecessary, cumbersome intravenous treatment, which may become a burden and reduce patient quality of life.

#### Conclusions

Evidence suggests that COPD in individuals with the PI*MZ genotype is mainly driven by smoking, and these individuals are only at a slightly higher risk of lung disease than equivalent smoking PI*MM individuals. There is, therefore, little rationale for treatment of PI*MZ patients to differ from that of similar PI*MM patients. Current guidelines do not recommend the treatment of PI*MZ individuals with AAT therapy due to the absence of specific evidence for use in this population showing that potential benefits outweigh safety risks. Sufficiently powered randomized controlled trials would be required to provide this evidence; however, there is little rationale to justify the establishment of such a study. As such, preventative measures, principally highlighting the risks of smoking and encouraging patients to stop or avoid starting smoking, may be the most effective treatment for individuals with the PI*MZ genotype. It is important to note that smoking cessation in COPD in general is a disease-modifying intervention [56]; lung function decline is known to stabilize after smoking cessation in the majority of COPD patients [57, 58].

## Discussion

The key factor that currently precludes the treatment of PI*MZ individuals with AAT therapy is the lack of evidence supporting the benefits of the augmentation. This lack of evidence is at least partially related to the potential for creating a substantial burden in terms of healthcare resources and medication supply, as well as the treatment-related burden of life-long intravenous infusions. While early identification of AATD is important to encourage lifestyle change, it remains to be determined whether lower levels of AAT, compared with normal levels seen in PI*MM individuals, are associated with a disease risk and emphysema/COPD progression, justifying the need for therapy. Current guidelines do not recommend use of AAT augmentation therapy in PI*MZ patients and only provide recommendations for therapy in patients with severe AATD (ZZ or Null genotypes) with emphysema [37–39]. Nonetheless, it remains a possibility that some individuals with the PI*MZ genotype and accelerated lung function decline may benefit from augmentation therapy; further investigation would be needed to determine the effects of treatment in these individuals. However, it is important to note that, at present, the lack of excessive lung function decline in patients should not in itself preclude the use of AAT therapy in severe disease (e.g., PI*ZZ individuals), and a holistic approach should be utilized; importantly, emphysema progression and morphology/distribution should be considered. This would seem to lessen the argument regarding the relevance of rapid lung function decline in PI*MZ individuals; however, it is unlikely that any patient experiences rapid decline consistently through life, with the detection of rapid decline possibly providing a ‘snapshot’ indicating an underlying issue that may warrant treatment. Development of future therapies, which could reduce the costs, lack of availability and cumbersomeness of augmentation therapy, will allow us to re-evaluate the paradigm regarding severe *vs*. non-severe AATD, and potentially expand our treatment goals in AATD.

Furthermore, it is important to note that while the Z allele is most commonly associated with AATD, this variant is only one of numerous variants that have been linked to AATD. Many rare/novel/null alleles are difficult to detect or are non-detectable by most testing methods (e.g., isoelectric focusing [IEF] and targeted polymerase chain reaction), and can only be detected by genetic sequencing, the availability of which is highly variable [59]. In particular, many rare alleles are M-like (with similar IEF banding pattern to the wild-type M protein). As such, many PI*MZ individuals with lower AAT levels and COPD may, in fact, be compound heterozygotes for the Z allele and a rare/M-like allele [59]. A recent study has reported a high rate of rare deficiency alleles in individuals who were previously identified as PI*MZ [60], which raises the question of what proportion of PI*MZ patients with severe disease/fast decline are ‘true’ PI*MZs. The challenges to accurately diagnosing rare/M-like Z compound heterozygotes include a lack of awareness and ability to detect rare alleles (e.g., with gene sequencing).

Although not the focus of the present paper and not relevant to the provision of AAT therapy, there is also increasing evidence that the PI*MZ genotype is a risk factor for liver disease in terms of clinically significant portal hypertension and low-grade liver fibrosis [61]. Published literature consistently identifies heterozygous PI*Z individuals to have increased risk for cirrhosis and liver failure requiring transplantation [62, 63]. Smaller retrospective studies suggest that a primary liver carcinoma might develop even in a heterozygote state of PI*Z AATD, even without concurrent liver disease [64]. Thus, it is important to recognize that the relevant problem in the PI*MZ population is not to prove their evidently lower risk of disease development in comparison to PI*ZZ individuals, but rather to understand their most likely increased risk of disease development in comparison to the population of AAT non-deficient individuals with the same risk factors. This additional risk supports the importance of identifying PI*MZ individuals in order to advise on preventative measures such as hepatitis vaccination, in addition to smoking cessation.

## Conclusions

Several points for and against the provision of AAT augmentation therapy in patients with the PI*MZ genotype have been presented (Table 1). Arguments discussed here do not suggest that augmentation therapy should be administered to PI*MZ patients, but it is clear that there is a gap in knowledge regarding the utility of AAT therapy in PI*MZ individuals. However, as the PI*MZ genotype is a relatively common risk factor for COPD, it can be agreed that the recognition and identification of the PI*MZ genotype is of ultimate importance, as lifestyle modifications can substantially influence the clinical course of disease. The provision of AAT-specific therapy to patients with this genotype would be dependent on further prospective studies focused on better understanding the natural history of PI*MZ AATD, and studies evaluating the response of PI*MZ individuals to augmentation therapy.

**Table 1:** Summary points for/against the working hypothesis regarding the use of AAT augmentation therapy in individuals with the PI*MZ genotype

Pro	Con
AAT deficiency in PI*MZ individuals represents an abnormal state that leads to a compromised response to inflammation and predisposition to a disease state; thus, in cases of poorly-controlled disease, correcting AAT deficiency may be beneficial	Augmentation therapy is not a cure, and is a life-long commitment; in PI*MZ individuals, there is little basis for potential clinical benefit to outweigh the burden of treatment
Additional genetic factors, such as ELANE expression, may contribute to a phenotype of PI*MZ individuals with greater protease/antiprotease imbalance and increased inflammation, potentially leading to rapid lung function decline	The only established risk modifier in PI*MZ individuals is smoking; smoking cessation should, therefore, be the focus of treatment
The prevalence of the PI*MZ genotype makes it a relatively common risk factor for COPD; the high prevalence along with high disease heterogeneity may suggest that general recommendations may not apply to each particular case and that there may be a subset of patients who could benefit from AAT augmentation therapy	The high prevalence of the PI*MZ genotype could create significant strain on the supply of a costly, plasma-derived therapy; the focus should be on improving the evidence for use of AAT augmentation therapy in PI*ZZ patients and increasing access to treatment for patients with severe AATD

*AAT* alpha-1 antitrypsin, *COPD* chronic obstructive pulmonary disease, *ELANE* elastase, neutrophil expressed, *NE* neutrophil elastase

## Data Availability

Not applicable.

## Abbreviations

AAT: Alpha-1 antitrypsin
AATD: Alpha-1 antitrypsin deficiency
COPD: Chronic obstructive pulmonary disease
CT: Computed tomography
ELANE: Elastase, neutrophil expressed
FEV_1_: Forced expiratory volume in 1 s
FVC: Forced vital capacity
NE: Neutrophil elastase
